# Association of renal function with mortality among hospitalized patients treated with remdesivir for COVID-19

**DOI:** 10.1371/journal.pone.0303896

**Published:** 2024-06-14

**Authors:** Maria Lourdes Gonzalez Suarez, Kristin C. Mara, Christina G. Rivera, Supavit Chesdachai, Evan Draper, Raymund R. Razonable

**Affiliations:** 1 Division of Nephrology and Hypertension, Department of Medicine, Mayo Clinic Rochester, Rochester, MN, United States of America; 2 Department of Quantitative Health Sciences, Mayo Clinic Rochester, Rochester, MN, United States of America; 3 Department of Pharmacy, Mayo Clinic Rochester, Rochester, MN, United States of America; 4 Division of Public Health, Infectious Diseases and Occupational Medicine, Department of Medicine, Mayo Clinic Rochester, Rochester, MN, United States of America; Monash University, AUSTRALIA

## Abstract

**Background and aim:**

Renal dysfunction is associated with poor outcomes in patients with coronavirus disease 2019 (COVID-19). In an effort to improve outcomes, intravenous remdesivir has been broadly used for the treatment of COVID-19 even in patients with low estimated glomerular filtration rate (eGFR). Our study assessed the residual risk of outcomes of patients with low eGFR despite treatment with remdesivir for COVID-19, during a timeframe prior to the expanded label across all levels of renal function.

**Methods:**

We conducted an observational, retrospective, multi-site cohort study of adults hospitalized with COVID-19 treated with at least one dose of remdesivir between November 6, 2020, and November 5, 2021. Electronic medical records were reviewed to obtain patient characteristics, related laboratory data, and outcomes. The primary endpoint was all-cause mortality by day 28. Multivariable logistic regression was used to evaluate association between groups.

**Results:**

The study population consisted of 3024 patients hospitalized with COVID-19 and treated with remdesivir. The median age was 67 [IQR 55, 77] years; 42.7% were women, and 88.6% were white. The median eGFR was 76.6 mL/min/1.73 m^2^ [IQR 52.5, 95.2]; the majority (67.2%) of patients had an eGFR ≥ 60, while 9% had an eGFR <30. All-cause mortality by day 28 was 8.7%. All-cause mortality rates were significantly higher among patients with impaired renal function (Odds Ratio [OR] 1.63 for patients with eGFR 30–59; OR 1.46 for eGFR 15–29; OR 2.42 for eGFR <15 and OR 5.44 for patients on dialysis) compared to patients with eGFR ≥60 mL/min/1.73m^2^.

**Conclusions:**

Lower eGFR remains an independent risk factor for mortality in COVID-19 even in patients treated with remdesivir.

## Introduction

Acute kidney injury (AKI) is common among patients with severe coronavirus disease 2019 (COVID-19). Signs of AKI include proteinuria, hematuria, and decreased glomerular filtration rate (GFR) manifested by an elevation of serum creatinine (SCr) levels or decreased urine output, including oliguria and/or anuria. AKI is associated with high mortality rates, especially among people requiring renal replacement therapy (RRT) [1].

Underlying chronic kidney disease (CKD) is recognized as one of the strongest risk factors associated with severe COVID-19 and subsequent poor outcomes [2]. Patients with CKD and end-stage kidney disease (ESKD) are particularly susceptible to acquire SARS-CoV-2 infection and develop severe COVID-19 likely as a result of their underlying medical comorbidities, including diabetes mellitus and hypertension [3].

Remdesivir is approved by the United States Food and Drug Administration for the treatment of COVID-19 in the United States, and since July 2023, this approval has been extended to patients with estimated glomerular filtration rate (eGFR) lower than 30 mL/min/1.73m^2^. Originally, these patients with impaired renal function were excluded from the clinical trials. One major concern for the use of intravenous remdesivir in patients with impaired renal function was the potential adverse drug toxicity related to the accumulation of remdesivir’s solubilizing excipient, sulfobutylether-β-cyclodextrin (SBECD), which is excreted by the kidneys [4]. However, the clinical benefits of the use of remdesivir in patients with reduced eGFR is suggested to outweigh the potential risk associated with SBECD [5]. Indeed, several studies have demonstrated that remdesivir was well tolerated in patients with CKD and ESKD [6, 7], although a few patients had transient transaminitis [8]. Recent data from a double-blind, randomized, placebo- controlled clinical trial showed evidence that remdesivir use was safe in patients with AKI and with advanced CKD (eGFR <30 mL/min/1.73m^2^) [9].

Clinical outcomes published data from real-world scenarios is limited on the use of remdesivir in patients with impaired renal function. In this study, we sought to assess this by comparing the clinical outcomes of hospitalized patients with different levels of renal function during their hospitalization for COVID-19.

## Methods

### Study design and patient population

This is a retrospective, multi-site cohort study of adults (age ≥18 years) who were hospitalized for the management of COVID-19 between November 6, 2020 and November 5, 2021 at Mayo Clinic Rochester and Mayo Clinic Health System (Midwest) and received treatment with at least one dose of remdesivir. At the time of this study period, remdesivir was not approved for patients with eGFR of < 30 mL/min/1.73m^2^, but our standard treatment protocol allowed for its off-label use during that time. Per Mayo Clinic standardized clinical practice, all patients hospitalized with COVID-19 were assessed for their eligibility for a 5-day intravenous remdesivir course. A centralized COVID-19 Treatment Review Panel evaluated all patients for their eligibility for the protocol of 5 days of remdesivir treatment. We excluded patients who received remdesivir prior to their hospitalization to Mayo Clinic Rochester and Mayo Clinic Health System (Midwest), and those who received other treatments such as convalescent plasma and anti-spike neutralizing monoclonal antibodies.

Electronic medical records were reviewed to obtain patient demographics and clinical characteristics including laboratory data, body mass index (BMI), World Health Organization (WHO) ordinal scale for disease severity (available as the maximum during admission), and Charlson comorbidity index. Data was retrieved via automated reporting and verified using manual review. WHO ordinal scale was modified for this study. Patients requiring low flow mask or nasal cannula were grouped as modified WHO scale 4.

No direct patient contact was performed for this study. The Mayo Clinic Institutional Review Board considered this study as exempt (IRB number 20–012975). Consent was waived for this study, but only those who have provided research authorization for medical record review were included.

### Outcomes

The primary endpoint was all-cause mortality, which was defined as death from any cause within 28 days of the first dose of remdesivir. These outcomes were determined by manual review of electronic medical records. Secondary endpoints were death related to COVID-19, and risk of all cause re-hospitalization and/or emergency department visit within 30 days of remdesivir initiation.

### Statistical analysis

Data were analyzed using median and interquartile range (IQR) for continuous variables and frequencies and percentages for categorical variables. Estimated GFR was calculated by CKD-EPI 2021. Creatinine values used to estimate GFR were more likely not on a steady-state due to ongoing acute kidney injury, and they were used as a pragmatic requirement for statistical analysis, with attendant limitations. Patients were categorized by eGFR in 5 groups: eGFR >60, eGFR 30–59, eGFR 15–29, eGFR <15 and patients on dialysis. Continuous data was compared between groups using a Kruskal-Wallis test, and categorical data was compared using Fisher’s exact test.

Multivariable logistic regression was used to evaluate the association between groups and death within 28 days after adjusting for other patient and clinical characteristics. In the event of missing data, data was not replaced. Rates of subsequent ED visits and hospital encounters have been calculated using the Aalen-Johansen method where death was considered a competing risk. Cox proportional hazards regression was used to assess the association between eGFR groups and subsequent ED visits/hospital encounters. Statistical analyses were performed using SAS version 9.4 software (SAS Institute, INC; Cary, NC).

## Results

During the one-year study period, a total of 3024 patients were hospitalized with COVID-19 received at least one dose of remdesivir. The median age was 67 years [IQR 55, 77]; 42.7% were women (n = 1293), and 88.6% were white (n = 2684). Hypertension (38.1%), cardiovascular diseases (33.6%), and diabetes mellitus (31.7%) were the most common medical comorbidities.

The median eGFR was 76.6 mL/min/1.73 m^2^ [IQR 52.5, 95.2]. The majority (67.2%) of patients had an eGFR ≥ 60 mL/min/1.73m^2^, while 9% (n = 273) had an eGFR <30 mL/min/1.73m^2^. Of those, 88 patients (2.9%) were dependent on dialysis.

While all patients received at least one dose of remdesivir, only 89.5% of patients (n = 2706) completed the full 5-day course of treatment. No patient received 10 days of remdesivir treatment.

There was no significant difference in treatment completion among groups according to their eGFR (p = 0.58). The median BMI was 31 kg/m2 [IQR 27, 37], and it was not significantly different across eGFR groups. Patients with an eGFR ≥60 mL/min/1.73m^2^ had significantly lower Charlson comorbidity index (I.e., less comorbidity) when compared with patients with lower eGFR (2 vs. 7–8, respectively; p<0.001) Table 1. According to the WHO Ordinal Scale for COVID-19 disease severity, there was a significant difference across the groups on the need for oxygen supplementation (p<0.001); patients with eGFR ≥60 mL/min/1.73m^2^ were significantly less likely to require any oxygen supplementation during their hospitalization (Table 2).

**Table 1:** Patient and treatment characteristics.

	Total (N = 3024)	Dialysis (N = 88)	eGFR < 15 (N = 26)	eGFR 15–29 (N = 159)	eGFR 30–59 (N = 718)	eGFR ≥ 60 (N = 2033)	p value
**Age (years)**, Median (IQR)	67 (55, 77)	66 (58, 72)	73 (66, 83)	76 (67, 84)	76 (67, 84)	62 (50, 73)	<0.001
**Gender**							0.044
Female	1291 (42.7%)	38 (43.2%)	15 (57.7%)	81 (50.9%)	320 (44.6%)	837 (41.2%)	
Male	1733 (57.3%)	50 (56.8%)	11 (42.3%)	78 (49.1%)	398 (55.4%)	1196 (58.8%)	
**Race/ethnicity**							<0.001
White	2680 (88.6%)	65 (73.9%)	24 (92.3%)	149 (93.7%)	667 (92.9%)	1775 (87.3%)	
American Indian/Alaskan Native	13 (0.4%)	2 (2.3%)	0 (0.0%)	2 (1.3%)	1 (0.1%)	8 (0.4%)	
Asian	59 (2.0%)	5 (5.7%)	1 (3.8%)	1 (0.6%)	9 (1.3%)	43 (2.1%)	
Black	70 (2.3%)	5 (5.7%)	0 (0.0%)	3 (1.9%)	15 (2.1%)	47 (2.3%)	
Hispanic/Latino	141 (4.7%)	7 (8.0%)	1 (3.8%)	3 (1.9%)	14 (1.9%)	116 (5.7%)	
Native Hawaii/Pacific Islander	4 (0.1%)	1 (1.1%)	0 (0.0%)	0 (0.0%)	0 (0.0%)	3 (0.1%)	
Other	20 (0.7%)	3 (3.4%)	0 (0.0%)	0 (0.0%)	4 (0.6%)	13 (0.6%)	
Unknown	37 (1.2%)	0 (0.0%)	0 (0.0%)	1 (0.6%)	8 (1.1%)	28 (1.4%)	
**BMI**							0.12
Median (IQR)	31 (27, 37)	30 (25, 36)	31 (22, 36)	32 (27, 38)	31 (26, 36)	31 (27, 37)	
**Charlson Comorbidity Index**, Median (IQR)	3 (1, 6)	8 (6, 10)	7 (4, 9)	7 (4, 9)	5 (2, 8)	2 (0, 4)	<0.001
**Modified WHO Scale at Remdesivir start**							<0.001
3 - hospitalized no O_2_	2288 (75.7%)	53 (60.2%)	17 (65.4%)	115 (72.3%)	549 (76.5%)	1554 (76.4%)	
4 – mask/nasal canula*	500 (16.5%)	21 (23.9%)	4 (15.4%)	30 (18.9%)	104 (14.5%)	341 (16.8%)	
5 - high flow noninvasive	197 (6.5%)	11 (12.5%)	5 (19.2%)	9 (5.7%)	50 (7.0%)	122 (6.0%)	
6 - Invasive	39 (1.3%)	3 (3.4%)	0 (0.0%)	5 (3.1%)	15 (2.1%)	16 (0.8%)	
**Days from admission to remdesivir initiation**, Median (range)	0 (0–16)	0 (0–12)	0 (0–1)	0 (0–9)	0 (0–13)	0 (0–16)	0.011
0	2048 (67.7%)	47 (53.4%)	16 (61.5%)	98 (61.6%)	497 (69.2%)	1386 (68.2%)	
1	855 (28.3%)	33 (37.5%)	10 (38.5%)	52 (32.7%)	186 (21.8%)	574 (28.2%)	
2+	121 (4.0%)	8 (9.1%)	0 (0.0%)	9 (5.7%)	35 (4.9%)	73 (3.6%)	
**Number of days on Remdesivir**							0.58
1	91 (3.0%)	3 (3.4%)	2 (7.7%)	3 (1.9%)	24 (3.3%)	59 (2.9%)	
2	80 (2.6%)	4 (4.5%)	1 (3.8%)	5 (3.1%)	17 (2.4%)	53 (2.6%)	
3	73 (2.4%)	3 (3.4%)	1 (3.8%)	5 (3.1%)	18 (2.5%)	46 (2.3%)	
4	74 (2.4%)	1 (1.1%)	1 (3.8%)	2 (1.3%)	13 (1.8%)	57 (2.8%)	
5/Completed	2706 (89.5%)	77 (87.5%)	21 (80.8%)	144 (90.6%)	646 (90.0%)	1818 (89.4%)	

*Based on our modified definition of WHO ordinal scale. Patients requiring low flow mask or nasal cannula were grouped as modified WHO scale 4.

**Table 2:** Outcomes according to eGFR.

	Total (N = 3024)	Dialysis (N = 88)	eGFR < 15 (N = 26)	eGFR 15–29 (N = 159)	eGFR 30–59 (N = 718)	eGFR ≥ 60 (N = 2033)	p value
**Modified WHO scale at discharge**							<0.001
3 - hospitalized no O_2_	464 (15.3%)	5 (5.7%)	2 (7.7%)	26 (16.4%)	110 (15.3%)	321 (15.8%)	
4 – mask/nasal canula*	1371 (45.3%)	37 (42.0%)	12 (46.2%)	66 (41.5%)	314 (43.7%)	942 (46.3%)	
5 - high flow noninvasive	828 (27.4%)	19 (21.6%)	8 (30.8%)	41 (25.8%)	183 (25.5%)	577 (28.4%)	
6 - Invasive	152 (5.0%)	7 (8.0%)	0 (0.0%)	6 (3.8%)	38 (5.3%)	101 (5.0%)	
7 - ECMO	4 (0.1%)	0 (0.0%)	0 (0.0%)	0 (0.0%)	0 (0.0%)	4 (0.2%)	
8 - Expired	186 (6.2%)	20 (22.7%)	4 (15.4%)	20 (12.6%)	67 (9.3%)	75 (3.7%)	
No data from metrics	19 (0.6%)	0 (0.0%)	0 (0.0%)	0 (0.0%)	6 (0.8%)	13 (0.6%)	
**Subsequent ED/Hospital Encounter (30d)** ^**a**^	141 (4.7%)	1 (1.1%)	0 (0.0%)	8 (5.0%)	46 (6.4%)	86 (4.2%)	0.049
**Death w/in 28 days from remdesivir start**	264 (8.7%)	22 (25.0%)	6 (23.1%)	22 (13.8%)	106 (14.8%)	108 (5.3%)	<0.001
**COVID death w/in 28 days**							
** Confirmed Covid-19-related death**	218 (7.2%)	6 (0.8%)	6 (23.1%)	21 (13.2%)	86 (12.0%)	87 (4.3%)	<0.001
** Possibly Covid-19-related death**	10 (0.3%)	0 (0.0%)	0 (0.0%)	0 (0.0%)	4 (0.6%)	6 (0.3%)	0.66
** No**	23 (0.8%)	4 (4.5%)	0 (0.0%)	1 (0.6%)	10 (1.4%)	8 (0.4%)	0.001
** Unknown**	13 (0.4%)	0 (0.0%)	0 (0.0%)	0 (0.0%)	6 (0.8%)	7 (0.3%)	0.40
**Death w/in 5 days from remdesivir start**	30 (1.0%)	1 (1.1%)	1 (3.8%)	4 (2.5%)	15 (2.1%)	9 (0.4%)	<0.001
**Death between days 6–28, conditioned on survival for first 5 days**	234 (7.8%)	21 (24.1%)	5 (20.0%)	18 (11.6%)	91 (12.9%)	99 (4.9%)	<0.001

^a^Rates have been calculated using the Aalen-Johansen method where death was considered a competing risk. Cox proportional hazards regression was used to compare groups.

*Based on our modified definition of WHO ordinal scale. Patients requiring low flow mask or nasal cannula were grouped as modified WHO scale 4.

### Primary outcome

**Fig 1:**
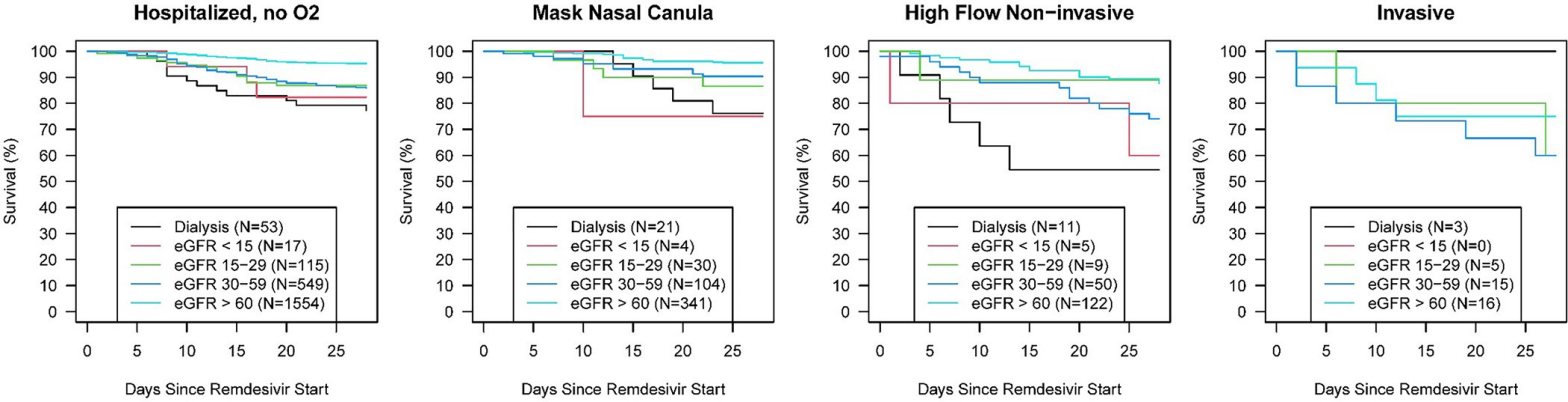
Survival rate according to eGFR and oxygen requirements.

Residual all-cause mortality within 28 days after receiving at least one dose of remdesivir was 8.7% (n = 264). Univariate analysis showed that overall mortality was significantly higher in patients requiring dialysis when compared with patients with eGFR ≥ 60 mL/min/1.73m^2^ (25% vs. 5.3%, respectively; p<0.001; Table 2). The majority of the deaths were due to COVID-19, and patients with eGFR<15 mL/min/1.73m^2^ were at higher risk of death when compared with patients with eGFR >60 (p<0.001) Table 3, (Fig 1).

**Table 3:** Univariate model of patient outcomes.

	Subsequent ED/Hospital Encounter		Death within 28 days of remdesivir initiation (all-cause)		COVID-19 Death within 28 days of remdesivir initiation	
	Hazard Ratio (95% CI)	p-value	Odds Ratio (95% CI)	p-value	Odds Ratio (95% CI)	p-value
**Group**						
Dialysis	0.52 (0.10–2.64)	0.43	4.31 (1.77–10.50)	0.001	5.75 (3.28–10.08)	<0.001
eGFR < 15	0.56 (0.03–9.19)	0.68	4.45 (1.01–19.66)	0.049	6.71 (2.63–17.13)	<0.001
eGFR 15–29	1.44 (0.71–2.93)	0.32	1.32 (0.47–3.73)	0.60	3.40 (2.05–5.65)	<0.001
eGFR 30–59	1.68 (1.17–2.42)	0.005	2.99 (1.93–4.64)	<0.001	3.04 (2.23–4.16)	<0.001
eGFR ≥ 60	Reference		Reference		Reference	
**c-statistic**	0.56		0.64		0.66	

After adjusting for age, gender, completion of remdesivir treatment and Charlson comorbidity index, patients with higher eGFR had higher odds of survival within 28 days when compared with patients with lower eGFR. Compared to those with normal renal function, the odds of residual all-cause mortality was significantly higher among patients with eGFR 30–59 (Odds Ratio: 1.63 [95% confidence interval: 1.19–2.22), and patients on dialysis (OR: 5.44 (95%CI: 3.02–9.77) Table 4).

**Table 4:** Multivariable models of patient outcomes.

	Subsequent ED/Hospital Encounter		Death within 28 days of remdesivir initiation (all-cause)		COVID death within 28 days of remdesivir initiation	
	Hazard Ratio (95% CI)	p-value	Odds Ratio (95% CI)	p-value	Odds Ratio (95% CI)	p-value
**Group**						
Dialysis	0.37 (0.07–1.90)	0.23	5.44 (3.02–9.77)	<0.001	5.58 (2.98–10.45)	<0.001
eGFR < 15	0.40 (0.02–6.72)	0.53	2.42 (0.85–6.87)	0.097	3.31 (1.17–9.40)	0.024
eGFR 15–29	1.14 (0.54–2.41)	0.72	1.46 (0.86–2.48)	0.16	1.95 (1.13–3.38)	0.017
eGFR 30–59	1.43 (0.96–2.12)	0.081	1.63 (1.19–2.22)	0.002	1.70 (1.21–2.39)	0.002
eGFR ≥ 60	Reference		Reference		Reference	
**Age (per 10 years)**	1.02 (0.91–1.15)	0.76	1.80 (1.60–2.02)	<0.001	1.73 (1.53–1.96)	<0.001
**Gender**						
Female	Reference		Reference		Reference	
Male	1.30 (0.92–1.84)	0.14	1.24 (0.94–1.63)	0.13	1.30 (0.96–1.75)	0.087
**Charlson Comorbidity Index (per 1 point)**	1.05 (1.00–1.10)	0.052	1.02 (0.98–1.06)	0.35	1.00 (0.96–1.05)	0.90
**Completed Remdesivir**	0.81 (0.48–1.36)	0.42	0.44 (0.31–0.64)	<0.001	0.48 (0.32–0.72)	<0.001
**Modified WHO Scale at Remdesivir start**	m1					
3 - hospitalized no O_2_	Reference		Reference		Reference	
4 –mask/nasal canula*	0.89 (0.55–1.42)	0.61	0.77 (0.52–1.14)	0.19	0.59 (0.37–0.93)	0.024
5 - high flow noninvasive	1.05 (0.54–2.04)	0.89	2.60 (1.69–4.00)	<0.001	2.74 (1.76–4.27)	<0.001
6 - Invasive	0.31 (0.02–5.07)	0.41	5.79 (2.66–12.61)	<0.001	3.55 (1.51–8.33)	0.004
**c-statistic**	0.61		0.78		0.78	

*Based on our modified definition of WHO ordinal scale. Patients requiring low flow mask or nasal cannula were grouped as modified WHO scale 4.

### Secondary outcomes

Table 3 lists the risks of COVID-19 related deaths as stratified by renal function. In a multivariable analysis Table 4, the odds of COVID-19 death increase with declining renal function. Compared to patients with normal renal function, the risk of death associated with COVID-19 was significantly highest among those on dialysis (OR: 5.58 [95% CI: 2.98–10.45] *p*<0.001). Even after adjusting for the modified WHO Ordinal Scale at the start of remdesivir treatment, the groups with impaired renal function are more likely to die within 28 days compared to those with normal renal function (Table 4). We assessed the interaction term between renal function groups and the modified WHO Ordinal Scale, to see if there were differences based on combination of the two or if they were independent predictors, and did not find a significant interaction between the two (p = 0.7).

All cause re-hospitalization and/or emergency department visits within 30 days of remdesivir initiation were observed in 4.7% of patients (n = 141). Univariate model showed that patients with an eGFR between 30 and 59 mL/min/1.73m^2^ had the highest risk of rehospitalization (Table 2). However, this did not appear to maintain its significance in multivariable model (Table 4).

## Discussion

This study reports two notable findings. First, despite treatment with remdesivir, there remains a high mortality from COVID-19 among patients with increasing impairment in renal function, especially those with eGFR < 60 mL/min/1.73m^2^ and on dialysis. Patients with impaired renal function are also at increased risk of more severe disease, as indicated by higher requirement of oxygen supplementation during their hospitalization. And second, this study observed no increased risk of hepatic toxicity during intravenous remdesivir treatment of COVID-19 among patients with impaired renal function.

Our study was conducted in 2020–2021, which spanned the period prior to and immediately after the availability of vaccines. At the time of this study, remdesivir was approved only for treatment of hospitalized patients with COVID-19. The mortality rate of 8.7% in our study reflects the high risk of mortality among those who qualified for remdesivir treatment at that time and allowed us to investigate the effect of different levels of renal function. Several comorbidities have been associated with worse outcomes in patients with COVID-19, including hypertension and diabetes, which are common comorbidities among patients with renal dysfunction [10]. In addition, AKI is also an independent risk factor for higher mortality among patients with COVID-19 [11]. Accordingly, there have been efforts to improve the outcomes by ensuring that intravenous remdesivir is administered for COVID-19 among high-risk patients, including those with impaired renal function. Despite this, however, our study demonstrates that the mortality rates of patients with impaired renal function remain higher compared to those with normal renal function. Our findings concur with several studies that included patients with eGFR <30 mL/min/1.73m^2^ in which there is an ongoing and unmet clinical need in this population, given their reported mortality rates of 21.4–55.9% despite treatment with remdesivir [12–15]. Efforts to prevent COVID-19 in these high-risk populations should be strongly encouraged and facilitated.

Subsequent to this study timeframe, the US FDA had approved the use of 3-day course of intravenous remdesivir for the early treatment of mild to moderate COVID-19 among high-risk patients in the outpatient setting. It is therefore important that all high-risk patients, especially those with renal dysfunction to get diagnosed early as soon as symptoms of COVID-19 occur. This is an effort to ensure they receive remdesivir or other antiviral drug treatment early to prevent progression to severe and critical illness [14]. Among patients who progressed to severe disease and require hospitalization, our previous study encouraged that they complete the recommended 5-day course of remdesivir (either inpatient or outpatient settings), since non-completion of treatment was significantly associated with a higher risk of mortality [16].

Among all patient groups, those requiring dialysis were at increased risk of more severe disease, as indicated the need for invasive oxygen supplementation during their COVID-19 hospitalization. In addition, those requiring dialysis also have the highest in-hospital all-cause mortality. Similar findings have been reported by others [17–20]. In general, patients on dialysis have a higher risk of mortality when compared to general population; however, once other comorbidities are adjusted, the independent association of CKD with a higher risk of mortality could be due to an impaired or altered immunological response to infections among patients on dialysis. Other considerations are the effects of hemodialysis on remdesivir and its metabolites [21, 22]. The current remdesivir package insert states that no dosage adjustment is required during dialysis. The REDPINE study will be able to provide additional pharmacokinetic data to address dosing in patients requiring dialysis [9]. No dose adjustment is required for patients requiring dialysis either due to ESKD or AKI at this time.

### Limitations

Our study has several limitations. It was a retrospective study of observational data from a review of electronic medical records. Remdesivir-treated patients were not compared to patients not treated with remdesivir, an analysis that would be challenging to perform outside of a prospective randomized controlled trial given confounders in which more ill patients may be more likely to be prescribed an antiviral treatment. Estimated GFR may not be an accurate representation in the acute setting to recognize those patients with CKD versus patients with severe AKI. Dialysis vintage was not included, preventing ability to identify those patients who were receiving temporary dialysis for AKI. There is a selection bias in the population that received remdesivir, as many of these patients were very sick, and while our institutional protocol allowed for the use of remdesivir in patients with renal dysfunction, some providers may have hesitated to provide this treatment for their patients with AKI and ESKD. This study was conducted over a span of one year, and included those patients prior to the availability of vaccines. The interpretation of this data should also take into account the circulating variants, which presumably are due to wild-type parent virus through alpha, beta, gamma and the delta variants of concern during the period of this study. Currently, Omicron subvariants are the predominant circulating strain worldwide. Finally, the total number of patients with impaired renal function, particularly those with GFR less than 15, was very low. The unexpected finding of lower risk of mortality among patients with modified WHO ordinal scale 4 (mask/ nasal cannula use) when compared with those not requiring supplemental oxygen (reference group) may be due to an unmeasured and unidentified confounder that was difficult to ascertain using our dataset. Future investigations on this finding are required.

## Conclusions

Our study showed that declining renal function was associated with elevated rates of death among patients hospitalized for COVID-19 despite treatment with intravenous remdesivir, suggesting an ongoing, unmet clinical need especially among this vulnerable population that may be mitigated by primary prevention measures including vaccination and treatment as early as feasible.

## Supporting information

S1 Data (XLSX)
